# Microbial Adhesion to Different Thermoplastic Denture Base Materials in Kennedy Class I Partially Edentulous Patients

**DOI:** 10.7759/cureus.60421

**Published:** 2024-05-16

**Authors:** Hassan M Sakr, Mahmoud R AbdulSalam, Mostafa I Fayad, Rania Moussa, Abdullah Ali H Alzahrani

**Affiliations:** 1 Department of Prosthodontic Dental Science, College of Dentistry, Al-Baha University, Al-Baha, SAU; 2 Department of Removable Prosthodontics, Faculty of Dentistry, Al-Azhar University, Cairo, EGY; 3 Department of Prosthodontics, Faculty of Dentistry, Horus University, New Damietta, EGY; 4 Department of Substitutive Dental Science, College of Dentistry, Taibah University, Madinah, SAU; 5 Department of Dental Health, School of Applied Medical Sciences, Al-Baha University, Al-Baha, SAU

**Keywords:** partially edentulous patients, kennedy’s class i, denture base, thermoplastic material, microbial adhesion

## Abstract

Introduction

Since the polished and fitting surface of the denture base may promote the colonization of microorganisms, it is essential to know how the different types of denture bases prevent or encourage the adhesion of microorganisms. This study aimed to compare the microbial adhesion to the polished and fitting surfaces of thermoplastic nylon, thermoplastic acetal, and thermoplastic acrylic denture bases in Kennedy Class Ⅰ, partially edentulous patients.

Materials and methods

Thirteen patients were included in the study. The group consisted of eight males (61.54%) and five females (38.46%), with an age range of 41-50 years (mean age 46.1 years). Three types (groups) of removable partial dentures will be made for each patient using different thermoplastic denture base materials: thermoplastic nylon; thermoplastic acetal; and thermoplastic acrylic. The polished and fitting surfaces of the denture bases were swabbed after a one-month follow-up period. Microbial adhesion was evaluated by counting the microorganisms' colony-forming units (CFU) in the collected specimens. The data were collected and statistically analyzed.

Results

The study revealed no statistically significant difference in microbial adhesion to both polished and fitting surfaces between all types of studied thermoplastic denture base materials. However, the results showed that for the polished surface, the microbial adhesion median of thermoplastic acrylic denture base (40.5 CC x 10^2^/ml) was higher than that of thermoplastic acetal (29.0 CC x 10^2^/ml) and thermoplastic nylon (16.0 CC x 10^2^/ml). Regarding the fitting surface, the microbial adhesion median of thermoplastic acrylic (51.0 CC x 10^2^/ml) is higher than that of thermoplastic acetal (41.0 CC x 10^2^/ml) and thermoplastic nylon (23.0 CC x 10^2^/ml).

Conclusion

The thermoplastic nylon denture base materials showed less microbial adhesion among the studied thermoplastic materials, so it may be recommended to be used as a denture base material for individuals at high risk of denture stomatitis.

## Introduction

Numerous studies have confirmed that tooth loss significantly impacts biological, social, psychological, and overall patient health [1,2]. Despite a significant decrease in the prevalence of tooth loss in many countries over the past few decades [3], the demand for removable prosthodontic treatment remains high, particularly for Kennedy Class I partially edentulous patients [4].

Removable partial denture prosthesis (RPD) rehabilitation is widely applied to Kennedy Class I and II partially edentulous patients. If the fabricated RPD does not follow biological and mechanical considerations, considerable damage might occur to the remaining hard and soft tissues [5].

Various materials can be utilized to fabricate RPD, most commonly cobalt-chromium alloys and polymethyl methacrylate resin [6]. Polymethyl methacrylate (PMMA) is widely used for prosthetic dental applications such as denture bases, obturators, and temporary or provisional crowns [7].

Recently, newer materials, known as "thermoplastic material" or "flexible," have been developed. Thermoplastic dentures are a highly regarded alternative to conventional hard-fitted dentures, as they provide enhanced patient comfort and improved esthetics through tooth-colored denture components. Furthermore, flexible dentures offer superior retention in distal extension cases by engaging the soft tissue undercuts and clasping the adjacent teeth [8].

The most commonly used thermoplastic materials in denture construction include thermoplastic acetal, thermoplastic nylon, and thermoplastic PMMA [9].

Microbial adhesion on the surface of dentures is a significant concern in dental care. The oral cavity provides an ideal environment for microbial colonization, and denture surfaces can easily harbor bacteria, fungi, and other microorganisms. Factors such as surface roughness, composition of the material, and surface free energy can contribute to the adhesion and growth of microbes [10, 11].

Microorganisms' colonization of denture surfaces makes it a potential source of oral pathogens that can form biofilms on different surfaces, resulting in the development of diseases such as dental caries, denture stomatitis (inflammation of the oral mucosa), inflammatory lesions, and other forms of oral infections, subsequently causing significant discomfort and pain for the patient wearing the dentures [12].

Epidemiological studies suggest that 11% to 70% of all patients wearing removable denture prostheses may suffer from denture stomatitis [13]. Microorganisms may also serve as reservoirs for disseminated infections with gastrointestinal and pleuropulmonary involvement [14].

Microorganisms' capacity to adhere to a removable prosthesis's polished tissue surfaces plays a pivotal role in the pathogenesis of denture stomatitis. This adhesion allows them to withstand the mechanical cleansing action of saliva and act as a focal point for further colonization [15].

Various approaches have been suggested for improving denture cleanliness; however, many of these techniques could potentially impact the properties of the denture base negatively, especially in cases where polymer-based materials are exposed to certain chemical disinfectants. This can lead to outcomes like increased surface roughness and diminished flexural strength [16-18]. One way to control microbial colonization is to construct the denture using a material that prevents or reduces microbial adhesion or retards microbial growth and biofilm formation [19].

To our knowledge, the previous studies compared the microbial adhesion between the conventional acrylic denture base with only one or two types of recently introduced thermoplastic denture base material [10,14,20-24]. So, the current study aimed to clinically compare the adhesion of microorganisms to the polished and fitting surface of the most commonly used thermoplastic materials in denture construction, namely thermoplastic nylon, thermoplastic acetal, and thermoplastic acrylic denture bases, using the same environmental conditions (in the same patient) in partially edentulous patients.

The null hypothesis was that the thermoplastic denture base material would not affect the adhesion of microorganisms to the polished and fitting surface of the removable partial dentures under the same environmental conditions.

## Materials and methods

The study was conducted at Al-Azhar University, Cairo, Egypt. The Faculty of Dentistry, Al-Azhar University, Ethics Committee reviewed and approved the study protocol (Ethical Application Ref: AUAREC2002208-12). The study was conducted for six months, from August 2023 to February 2024. Previous studies have determined that a sample size of 12 cases is sufficient to conduct the research with a statistical power of 0.80, a confidence interval of 0.95, and an alpha level of 0.05 [10,14,25]. A higher sample size calculation was considered (n=14). To allow for the possibility of participants dropping out due to illness, death, or difficulty with the research protocol.

Patients were selected from the outpatient clinic of the Removable Prosthodontics Department, Faculty of Dental Medicine, Cairo, Al-Azhar University. The participants agreed to follow the recommendations and denture hygiene instructions, and they shared in the study only after the explanation of study procedures and written informed consent were signed.

Patients with Kennedy Class I partially edentulous mandible opposing fully dentate maxilla were randomly selected. All of the chosen patients fulfilled the following criteria: they aged between 40 and 50 years, they had healthy, firm mucoperiosteum without any signs of inflammation or flabby tissues, and the oral salivary secretions were within the average amount and consistency of "thin viscid film. They also had no history of antibiotic intake for the past three weeks. Smokers, patients with systemic diseases, or patients using drugs that may affect the health of oral tissues were excluded.

The study design is a cross-over randomized clinical study, which means that all selected participants wear all dentures of the studied denture bases in a random sequence for one month for each denture. During the study period, the patients were instructed to follow the standard denture care guidelines, which consisted of mechanical brushing with water and overnight storage [18].

Three removable partial dentures were made for each patient: thermoplastic nylon (Acron®, Thermoplastic Nylon, Roko®, Poland) and thermoplastic acetal (Biocetal®, Thermoplastic Acetal, Roko®, Poland) partial dentures were made according to the injection molding technique [26], while the thermoplastic acrylic (Versacryl®, Thermoplastic Acrylic Industries, USA) was made according to the compression molding technique [27]. Each patient used each denture alternately for one month.

The patients were divided into two groups based on their age to evaluate any difference in microbial adhesion based on the patient's age. Group 1: Patients aged between 40 and 45 years. Group 2: Patients aged between 46 and 50 years.

**Figure 1:**
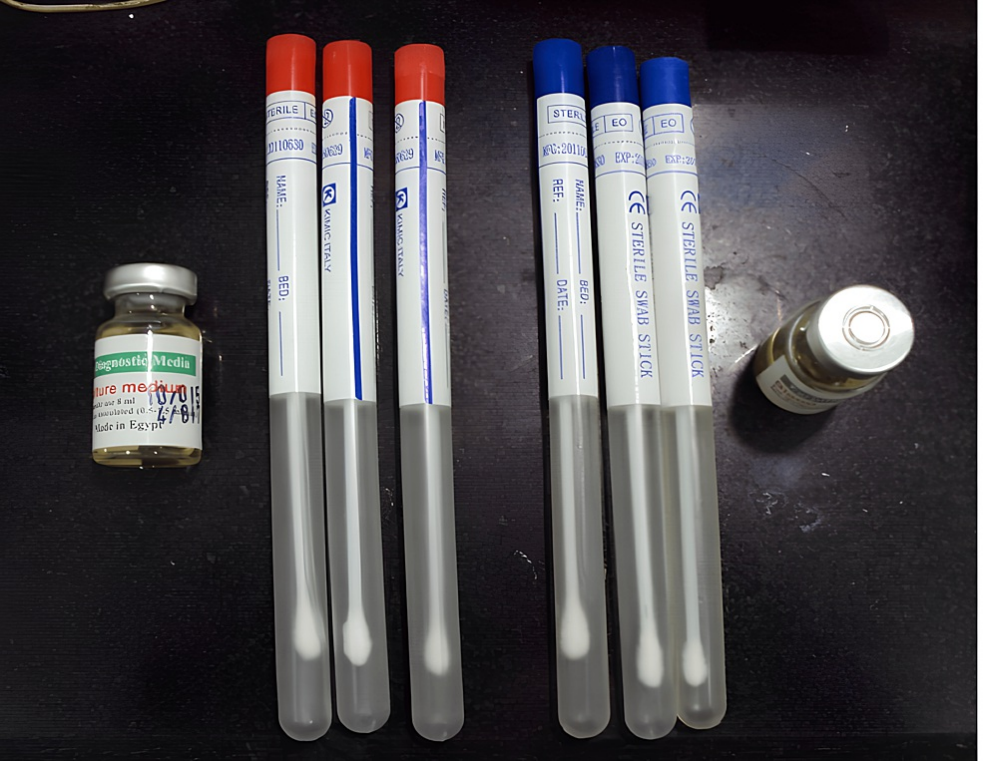
Sterile swab sticks and blood diagnostic medium (culture medium) used in the study

**Figure 2:**
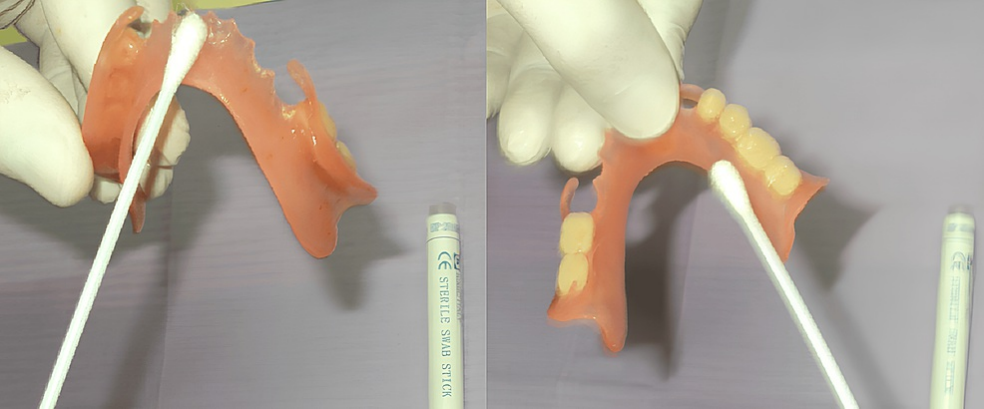
A swab taken from the fitting and polished surfaces of the denture

One month after the insertion of each denture, swabs were collected using a sterile swap stick (KIMIC Co., Italy) (Figure 1) by vigorous rubbing of the polished and fitting surfaces of the dentures (Figure 2).

**Figure 3:**
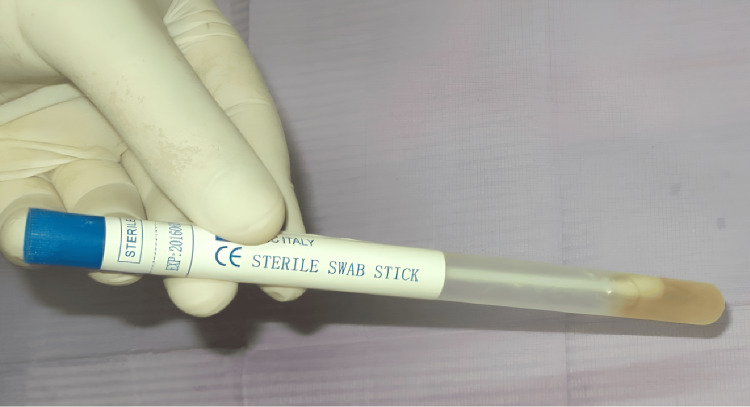
A swab stick sample inserted into the sterile plastic tube with the culture medium inside

The test tube was prepared by aspiration of 3 ml of blood diagnostic medium (EDM® Egyptian Diagnostic Media, Blood Diagnostic Media for Bacterial Culture, Egypt) by a sterile syringe from a blood diagnostic vial and added to the plastic sterile swab tube. Samples were inoculated immediately in a sterile glass tube containing 3 ml of blood culture medium. The tube was labeled with the patient's name, the type of denture base material, and the surface of the denture to be tested (Figure 3).

Around 100μL of each dilution was spread onto the surface of blood agar (Egyptian Diagnostic Media, EDM) and examined by an optical microscope (Olympus BX51® Fluorescence Microscope, Optical Microscope, Japan) at X400 magnification.

Microbial counts in different samples were calculated (using the plate counting method) by counting the colony-forming units (CFUs) on the blood agar plates [14].

Statistical analysis

The data were collected and statistically analyzed using IBM SPSS Statistics for Windows, Version 25 (Released 2017; IBM Corp., Armonk, New York, United States). The data were tested for normality by the Shapiro-Wilk test. Quantitative data were expressed as minimum, maximum, and median. A one-way analysis of variance (ANOVA) test was used to compare microbial adhesion among the three studied denture base materials. The significance of the obtained results was judged at the 5% level.

## Results

The study assessed the microbial adhesion to the polished and tissue surfaces of removable partial dentures made from thermoplastic nylon, thermoplastic acetal, and thermoplastic acrylic.

One participant dropped out, resulting in completion by 13 patients (n=13). The group consisted of eight males (61.54%) and five females (38.56%), with an age range of 41-50 years (mean age 46.1 years).

The results of the Kolmogorov-Smirnov and Shapiro-Wilk tests, which were used to assess the normality of the data, showed that the data were normally distributed, as illustrated in Table 1.

**Table 1:** Tests of normality *: This is a lower bound of the true significance; a: Lilliefors significance correction

	Kolmogorov-Smirnov^a^			Shapiro-Wilk		
	Statistic	df	Sig.	Statistic	df	Sig.
NylonP	.187	13	.200^*^	.887	13	.089
NylonF	.208	13	.127	.871	13	.054
AcetalP	.220	13	.085	.935	13	.398
AcetalF	.144	13	.200^*^	.933	13	.370
AcrylicP	.158	13	.200^*^	.942	13	.485
AcrylicF	.109	13	.200^*^	.963	13	.796

The assessment of microbial adhesion showed no significant differences among all studied materials for thermoplastic denture bases on polished and tissue surfaces (Table 2).

**Table 2:** Microbial adhesion comparison between the three studied denture base materials SD: standard deviation; F: F for one way analysis of variance (ANOVA) test; t: student t-test; p: p-value for comparison between the three studied denture base materials on each side; p0: p-value for comparison between polished and fitting surfaces for each material; *: the mean difference is significant at the 0.05 level

Microbial adhesion C.C. x10^2^/ml	G1 thermoplastic nylon (n=13)	G2 thermoplastic acetal (n=13)	G3 thermoplastic acrylic (n=13)	F	p
Polished					
Mean ± SD.	21.9 ± 15.5	33.2 ± 17.6	39.1 ± 23.6	2.687	0.082
Median (Min. – Max.)	16 (6 – 54)	29 (4 – 68)	40.5 (8 – 89)		
Fitting					
Mean ± SD.	32.2 ± 20.8	43.9 ± 21.8	51.9 ± 23.4	2.626	0.086
Median (Min. – Max.)	23 (10 – 79)	38 (18 – 90)	51 (20 – 95)		
T	1.420	1.368	1.380		
p_0_	0.169	0.184	0.180		

Regarding the polished surface, no statistically significant differences (p=0.082) were observed among all the examined denture base materials. Nevertheless, findings indicated that the median microbial adhesion of thermoplastic acrylic (40.5 × 10²/ml) was higher compared to that of thermoplastic acetal (29.0 × 10²/ml) and thermoplastic nylon (16.0 × 10²/ml).

Regarding the fitting surface, no significant differences (p=0.086) were observed across all types of denture bases examined. Nevertheless, the research found that thermoplastic acrylic had a higher median microbial adhesion (51.0 CC x 10^2^/ml) compared to both thermoplastic acetal (41.0 CC x 10^2^/ml) and thermoplastic nylon (23.0 CC x 10^2^/ml).

The microbial adhesion was compared between male and female patient groups (Table 3). The findings revealed a statistically significant difference between the two groups. Male patients exhibited lower microbial adhesion across all tested denture base materials compared to female patients.

**Table 3:** Microbial adhesion comparison between male and female patients for each studied denture base material SD: standard deviation; t: student t-test; p: p-value for comparison between male and female; *: statistically significant at p≤0.05

		Sex		t	p
		Male (n=8)	Female (n=5)		
Polished	G1 nylon				
	Mean ± SD.	11.6 ± 4.8	38.4 ± 11.4	5.943^*^	<0.001^*^
	Median (Min. – Max.)	11 (6 – 20)	38 (24 – 54)		
	G2 acetal				
	Mean ± SD	23 ± 9.3	49.6 ± 15.2	3.952^*^	0.002^*^
	Median (Min. – Max.)	26.5 (4 – 32)	50 (30 – 68)		
	G3 acrylic				
	Mean ± SD.	24.8 ± 12.5	62 ± 18.3	4.379^*^	0.001^*^
	Median (Min. – Max.)	22.5 (8 – 45)	60 (44 – 89)		
Fitting	G1 nylon				
	Mean ± SD	18.9 ± 5.6	53.4 ± 18.2	4.128^*^	0.011^*^
	Median (Min. – Max.)	19 (10 – 28)	52 (32 – 79)		
	G2 acetal				
	Mean ± SD	30 ± 9.1	66 ± 16.7	5.086^*^	<0.001^*^
	Median (Min. – Max.)	30 (18 – 44)	64 (48 – 90)		
	G3 acrylic				
	Mean ± SD	36.9 ± 13.4	75.8 ± 13	5.163^*^	<0.001^*^
	Median (Min. – Max.)	35 (20 – 58)	72 (62 – 95)		

The microbial adhesion was assessed among various age groups (Table 4). Group (a) comprised patients aged 41 to 45 years, while group (b) consisted of patients aged 46 to 50 years. The findings indicated no statistically significant differences among the different age groups.

**Table 4:** Microbial adhesion comparison between the two studied age groups SD: standard deviation; t: student t-test; p: p-value for comparison between the two studied groups of age

		Age (years) Group (a) Group (b)		t	p
		41 – 45 (n = 6)	46 – 50 (n = 7)		
Polished	G1 nylon				
	Mean ± SD	25.8 ± 19.5	18.6 ± 11.7	0.830	0.424
	Median (Min. – Max.)	22 (6 – 54)	14 (8 – 38)		
	G2 acetal				
	Mean ± SD	34.7 ± 25.1	32 ± 9.5	0.246	0.814
	Median (Min. – Max.)	31 (4 – 68)	28 (24 – 50)		
	G3 acrylic				
	Mean ± SD	45.7 ± 31.1	33.5 ± 15.2	0.920	0.377
	Median (Min. – Max.)	46 (8 – 89)	27 (18 – 60)		
Fitting	G1 nylon				
	Mean ± SD	37.5 ± 27.4	27.6 ± 13.8	0.846	0.415
	Median (Min. – Max.)	30 (10 – 79)	22 (16 – 52)		
	G2 acetal				
	Mean ± SD	49 ± 28.8	39.4 ± 14.5	0.777	0.453
	Median (Min. – Max.)	46 (18 – 90)	36 (24 – 64)		
	G3 acrylic				
	Mean ± SD	56.8 ± 30.2	47.6 ± 17.1	0.695	0.501
	Median (Min. – Max.)	60 (20 – 95)	44 (28 – 72)		

## Discussion

Microbial attachment to the oral epithelium, soft denture lining, and denture base materials has been extensively investigated in numerous in vitro and simulated environments. [20,22,24].

Limited in vivo research has been documented on this topic. Therefore, this study was conducted to evaluate the impact of denture base material on microbial adhesion to the polished and fitting surfaces of removable partial dentures. The findings indicated that no significant differences were observed in microbial adhesion among the various denture base materials studied, thus supporting the acceptance of the null hypothesis.

As dentures are not sterile and are used at the body‐external environment interface, it is possible to be colonized by microorganisms. Once placed in the patient's mouth, the denture surface becomes coated with an 'acquired pellicle' of salivary glycoproteins (including salivary amylase, albumin, mucin, and lysozyme) and immunoglobulins [19]. The prosthesis surfaces represent the interface being colonized by oral biofilm a few hours after placement [28].

The study focused on microbial adhesion rather than bacterial colonization, assessing how likely denture-based materials are to adhere to microorganisms, which is defined as the tendency of a denture-based material to adhere to the microorganisms [20].

The study evaluated the microbial adhesion in the same environmental conditions as it was conducted in the same patient to avoid any difference that may result from variation of salivary secretion composition or other oral environment factors across different individuals.

As there are complex and numerous interactions between the individual (age, health), their denture (age, material, hygiene/cleaning regime), patient dentulous status, and the resulting colonizing microorganisms [29], the inclusion criteria take into consideration all of these factors to avoid any bias affecting study outcomes.

A blood agar medium was utilized due to its non-selective nature and ability to support the growth of a wide variety of microorganisms [30].

As many clinical studies have confirmed, the adaptation process to new dentures is completed within a month [31]. Therefore, microbial adhesion in this study was assessed after one month of denture placement in order to ensure that the patient could comfortably wear the denture as part of their daily routine.

A special emphasis on oral hygiene instructions was given to patients. The patients were instructed to follow the standard denture care guidelines to minimize microbial buildup on the dentures [32].

The current study showed that the microbial adhesion to the fitting surface of the thermoplastic acrylic denture base was higher compared to the microbial adhesion to the fitting surfaces of the nylon and acetal denture bases, which may be due to the role of surface properties such as surface-free energy and surface roughness, which result in higher microbial detachment forces [14].

This result may be attributed to the difference in surface roughness among the different thermoplastic denture bases. As reported by Mekkawy et al. [33], they found significantly less surface roughness in the thermoplastic polyamide-nylon, followed by acetal, and the highest in the thermoplastic acrylic denture base.

Furthermore, the surface of acetal resin may carry a negative charge, which can help absorb salivary defense molecules such as statins and defensins. One study indicates that charged acrylic resins show anti-microbial activity, depending on the dosage [14]. Further studies are recommended to understand how microorganisms adhere to polymeric biomaterials, as it is essential to have a comprehensive understanding of the surface characteristics of biomaterials and microorganisms.

The results of this study agreed with the results of Ahmad et al., who found that C. albicans has a lesser opportunity to adhere to thermoplastic nylon than to acrylic resin denture base materials [34].

Sundari et al. [35] found that the heat-cured acrylic resin has a higher Candida albicans colony count than thermoplastic nylon resin after immersion in Ulee Kareng coffee (Coffea robusta).

This may be due to the bacterial adhesion being more likely on hydrophilic surfaces showing high values of surface free energy than on hydrophobic surfaces. For polymers, values of water contact angles between 40° and 70° are reported as most suitable for cell adhesion. In terms of the critical surface energy of acrylic materials, the zone of good adhesion is located at values greater than 40 mJ/m2 [11].

Ahmed et al. [36] studied microbial colonization on different polymeric denture base materials; they found that the flexible dentures show lesser Candida albicans colonization on upper denture fitting surfaces compared to conventional heat-cured acrylic resin and nylon-based dentures.

Karam et al. [10] evaluated the microbial adhesion and secretory immunoglobulin A (sIgA) levels in patients with different denture base materials. The study results showed that the flexible denture bases had less microbial adhesion on their fitting surfaces than conventional denture bases. They concluded that flexible dentures are more hygienic and biologically compatible than conventional dentures.

Developing new materials capable of controlling microbial adhesion could be significantly aided by knowledge of the complex interactions between oral microorganisms and resin-based materials.

The results showed that the microbial adhesion to the fitting and polished surfaces was higher in female patients than in male patients. Studies have shown that differences exist in the colonization of Candida species on denture surfaces between genders. Female denture wearers, especially those with complete maxillary dentures, tend to experience chronic atrophic candidiasis more frequently [37]. Moreover, non-Candida albicans Candida (NCAC) colonization on denture surfaces has been observed to differ between male and female denture wearers [38].

The microbial adhesion differences between male and female patient groups can be attributed to a combination of factors such as immune responses, microbial compositions, and microbiome dynamics. Understanding these sex-based variances is crucial for treatment strategies tailored to the specific needs of male and female patients.

The study has limitations, such as using the manual colony counter for colony counting instead of the digital colony counter due to its feasibility, so the exact microbial load might not have been estimated. However, the study gives an insight into the possible count of microbial contamination with different denture base materials. The present study evaluated microbial adhesion only in healthy, partially edentulous patients free of oral and systemic diseases. However, the effect of systemic disease or drugs taken on the microbial contamination of dental prostheses remained unknown. 

Further studies may also be needed to assess the impact of different saliva components, such as electrolytes, immunoglobulins, and enzymes, on microbial adhesion in both healthy and debilitated individuals. Moreover, studies need to delve into understanding how microorganisms adhere to polymeric biomaterials since a comprehensive understanding of biomaterial surface characteristics and microorganism behavior is crucial.

## Conclusions

Within the limitations of this study, the thermoplastic materials used for denture construction (acetal, nylon, and polymethylmethacrylate) exhibited no statistically significant difference in microbial adhesion when subjected to identical environmental conditions in patients wearing Class I partial dentures and adhering to standard denture care protocols. However, due to its comparatively lower values of microbial adhesion compared to other assessed thermoplastic denture base materials, thermoplastic nylon is recommended as an appropriate choice for denture base material in short-span bounded saddles for individuals at high risk of infection.
